# Calcium binding and permeation in TRPV channels: Insights from molecular dynamics simulations

**DOI:** 10.1085/jgp.202213261

**Published:** 2023-09-20

**Authors:** Chunhong Liu, Lingfeng Xue, Chen Song

**Affiliations:** 1 Center for Quantitative Biology, Academy for Advanced Interdisciplinary Studies, Peking University, Beijing, China; 2 Peking-Tsinghua Center for Life Sciences, Academy for Advanced Interdisciplinary Studies, Peking University, Beijing, China

## Abstract

Some calcium channels selectively permeate Ca^2+^, despite the high concentration of monovalent ions in the surrounding environment, which is essential for many physiological processes. Without atomistic and dynamical ion permeation details, the underlying mechanism of Ca^2+^ selectivity has long been an intensively studied, yet controversial, topic. This study takes advantage of the homologous Ca^2+^-selective TRPV6 and non-selective TRPV1 and utilizes the recently solved open-state structures and a newly developed multisite calcium model to investigate the ion binding and permeation features in TRPV channels by molecular dynamics simulations. Our results revealed that the open-state TRPV6 and TRPV1 show distinct ion binding patterns in the selectivity filter, which lead to different ion permeation features. Two Ca^2+^ ions simultaneously bind to the selectivity filter of TRPV6 compared with only one Ca^2+^ in the case of TRPV1. Multiple Ca^2+^ binding at the selectivity filter of TRPV6 permeated in a concerted manner, which could efficiently block the permeation of Na^+^. Cations of various valences differentiate between the binding sites at the entrance of the selectivity filter in TRPV6. Ca^2+^ preferentially binds to the central site with a higher probability of permeation, repelling Na^+^ to a peripheral site. Therefore, we believe that ion binding competition at the selectivity filter of calcium channels, including the binding strength and number of binding sites, determines Ca^2+^ selectivity under physiological conditions.

## Teaser

Using a recently developed multisite ion model, Liu et al. conducted molecular dynamics simulations to study the Ca^2+^ binding and permeation in TRPV channels. The ion binding strength and number of binding sites within the selectivity filter are found to be key factors determining the permeation patterns and probably ion selectivity.

## Introduction

Ion channels are important regulators of intracellular and extracellular ion concentration. The opening of ion channels generates ionic currents, which are fundamental signals in the nervous systems of animals (Hodgkin and Huxley, 1952). The selectivity of ion channels enables them to allow specific types of ions to pass through, which is a key feature of their biological function. For example, selective voltage-gated sodium channels (Na_V_ channels) and voltage-gated potassium channels (K_V_ channels) in neuronal membranes are involved in action potential generation and conduction. These channels respond to changes in membrane potential and generate currents specifically conducted by Na^+^ or K^+^. If these channels lose their selectivity, inward sodium and outward potassium currents cannot be generated and action potentials cannot spread among neurons (Abdul Kadir et al., 2018). Divalent ion channels, such as calcium channels that are weakly to highly selective for Ca^2+^, are also involved in essential physiological processes, including muscle contraction, neurotransmitter release, and hormone secretion (Ebashi and Endo, 1968; Ghosh and Greenberg, 1995; Wiederkehr et al., 2011). Some calcium channels are highly selective. For example, Ca_V_ channels can reach a selectivity of P_Ca_:P_Na_ ∼ 1,000 (ion permeability ratio) despite the high concentration of sodium in the extracellular environment. How ion channels distinguish between ions with subtle differences and selectively permeate specific types of ions are fundamental scientific questions in the field of ion channel research.

For ion channels with available structures, molecular dynamics (MD) simulation is a powerful method for studying the microscopic mechanism of ion permeation and selectivity. There has been much success using this method to elucidate the permeation and selectivity mechanism of monovalent cation channels, such as potassium and sodium channels (Bernèche and Roux, 2001; Jensen et al., 2010; Corry and Thomas, 2012; Xia et al., 2013; Köpfer et al., 2014; Flood et al., 2018; Kopec et al., 2018). Dehydration of the first solvation shell of cations and interactions between multiple ions are thought to be pivotal in the selectivity mechanism of these monovalent ion channels (Mironenko et al., 2021). For calcium channels, multiple hypotheses have been proposed to explain their selectivity (Boda et al., 2000; Corry et al., 2001; Sather and McCleskey, 2003). In the early 1980s, Tsien proposed that calcium channels achieve selectivity by forming multiple binding sites that have a higher affinity for Ca^2+^ than other ions in the pore. Ca^2+^ has been proposed to permeate in a knock-off manner, which requires a repulsive force from a second ion to facilitate the detachment of Ca^2+^ from the binding site (Tsien et al., 1987). In the meanwhile, the knock-on permeation mechanism has also been widely discussed in the field of ion channel research, which describes the phenomenon that multiple ions line up to move in a concerted manner within the pore. The two terms are different, with the former focusing on the dissociation of the bound ion and the latter focusing on the collective motion, both of which resulted from ion knocking. Atomistic and dynamic details are required to discuss the difference or consistency between the two. However, MD studies on calcium channels have been hindered by the lack of accurate calcium models in classical MD.

TRPV channels (transient receptor potential cation channel subfamily V) are excellent candidates for investigating calcium channel selectivity. The TRPV family has six members, and their structures share a similar tetramer scaffold but have distinct calcium selectivity (Venkatachalam and Montell, 2007). Among these, only TRPV5 and TRPV6 are thought to be Ca^2+^-selective (P_Ca_:P_Na_ > 100). Other members of this family, including the capsaicin receptor and thermosensor TRPV1, are considered non-selective cation channels (P_Ca_:P_Na_ < 10; Owsianik et al., 2006). Furthermore, the open-state structures of Ca^2+^-selective TRPV6 and non-selective TRPV1 are available (Cao et al., 2013; McGoldrick et al., 2018), providing great opportunities to investigate their ion binding, permeation, and selectivity using MD simulations. In closed-state crystal rTRPV6 structures, three Ca^2+^ binding sites in the selectivity filter (SF) were identified, formed by D541, T539, and M570, respectively (Saotome et al., 2016), which was confirmed by MD simulations (Sakipov et al., 2018). In the open-state cryoEM hTRPV6 structure, only one Ca^2+^ binding site near D542 in the middle of the SF was identified (McGoldrick et al., 2018). The constriction site in the SF of TRPV1 is formed by a simple glycine and has a rather spacious vestibule above it. Compared with non-selective TRPV1, Ca^2+^-selective TRPV6 has a longer and narrower SF formed by four residues, ^539^TIID^542^. How the differences in SF structures between TRPV6 and TRPV1 determine their distinct Ca^2+^ selectivity remains to be elucidated.

In this study, we conducted MD simulations for TRPV1 and TRPV6 using a recently developed multisite Ca^2+^ model (Zhang et al., 2020), which is more accurate in describing interactions between Ca^2+^ and proteins and has been successfully used to simulate multiple Ca^2+^ channels (Liu et al., 2021; Zhuang et al., 2022 *Preprint*; Schackert et al., 2023) to reveal the ion binding and permeation mechanisms through TRPV channels. Our results showed that despite the similar overall protein scaffolds, the differences in the SF structure resulted in distinct ion binding and permeation patterns in TRPV6 and TRPV1. The binding competition and the number of binding sites in SF could determine Ca^2+^ selectivity under physiological conditions.

## Materials and methods

### MD simulations of TRPV channels

The open-state structure of hTRPV6 (PDB ID: 6BO8; McGoldrick et al., 2018) used in this study was solved using cryo-EM at a resolution of 3.6 Å. The missing loop between S2 and S3 in this structure was modeled using Modeller (Šali and Blundell, 1993), utilizing the existing open structures (PDB ID: 6BO8, 7K4A; Bhardwaj et al., 2020) as the template. The whole protein structure and two truncated structures were used to build the protein–membrane simulation systems, retaining the channel pore domain (residue 475–588) and transmembrane domain (residue 317–608), respectively. For TRPV1, the DkTx (double-knot toxin)/RTX (resiniferatoxin)-bound open-state structure solved using cryo-EM at a resolution of 2.95 Å was used (Gao et al., 2016). A truncated structure containing only the transmembrane domain (residue 423–713) was used for the simulations.

A POPCbilayer in the simulation system was built using the CHARMM-GUI Membrane Builder (Wu et al., 2014). Ions were added to the system to reach different target concentrations using the CHARMM sodium model and the multisite calcium model developed by us recently (Zhang et al., 2020). The multisite calcium model was optimized for accurate calculation of calcium–protein interactions, and a recent QM/MM study further confirmed its reliability (Schackert et al., 2023). The ion concentration is the same on both sides of the membrane. The simulation systems were equilibrated using the standard CHARMM-GUI equilibration protocol before the production simulations (Jo et al., 2008), as summarized in Table S1. For the TRPV6 simulation system with the pore domain only, the system size was 9.76 × 9.76 × 9.55 nm^3^ and the thickness of the POPC bilayer (the distance between layers of P atoms) was about 3.7 nm. For the TRPV6 simulation system with the transmembrane domain, the system size was 10.62 × 10.62 × 11.07 nm^3^ and the thickness of the POPC bilayer was about 3.6 nm. For the TRPV1 simulation system with the transmembrane domain, the system size was 11.20 × 11.20 × 9.80 nm^3^ and the thickness of the POPC bilayer was about 3.6 nm. A transmembrane potential of 500 mV from the extracellular side to the cytosolic side was applied by adding an external constant electric field *E*, whose values were obtained by dividing the transmembrane voltage 500 mV with the simulation box length in the z direction. Such a high voltage is not ideal for simulating physiological conditions but is necessary for obtaining enough permeation events within accessible simulation time to get better statistics for now. All simulations were performed using the CHARMM36m force field (Huang et al., 2017) and the TIP3P water model with a time step of 2 fs. The v-rescale thermostat (Hess et al., 2008) with a time constant of 0.5 ps and the Parrinello–Rahman pressure coupling (Parrinello and Rahman, 1981) with a time constant of 5 ps were used to maintain the temperature and pressure at 310 K and 1.0 bar during the simulations. The particle-mesh Ewald method (Darden et al., 1993) was used to calculate electrostatic interactions. The van der Waals interactions were smoothly switched off from 1.0 to 1.2 nm. Gromacs 2018.6 was used to run the MD simulations and trajectory analysis (Abraham et al., 2015). Three independent 500-ns MD trajectories were collected for each simulation condition.

Due to the oversimplification of the simulation system, such as the single-component membrane model and the absence of ligand, the open-state structures of both TRPV1 and TRPV6 were found to be unstable in our MD simulations. Therefore, to maintain the open-state conformation, harmonic position restraints were applied to the majority of the α-carbon atoms of the protein with a force constant of 1,000 kJ mol^−1^ nm^−2^. While this might potentially limit the protein’s dynamics, it offers the advantage of faster convergence, which adequately serves our purpose: analyzing ion permeation through the particular open-state conformation. Still, some regions in the SF were kept free without restraints in the simulations. In simulations with the pore domain of TRPV6, the pore loop–forming SF (residue 539–542) and pore helix regions (residue 514–552) were free. In simulations with the transmembrane domain of TRPV6, the loops between the transmembrane helices and pore helix regions were free (residue 350–378, 405–424, 447–449, 470–475, 514–552). In TRPV1 simulations, the loop formed SF (residue 643–656) and the loops between transmembrane helices and pore helices (residue 456–468, 501–510, 533–537, 557–561, 600–656) were free. All the position restraints that are tested are summarized in Table S2.

### Umbrella sampling

We used umbrella sampling to calculate the potential of mean force (PMF) of Ca^2+^ and Na^+^ in TRPV1 and TRPV6. Systems with only one Ca^2+^/Na^+^ were prepared to calculate one-ion PMF. We applied flat-bottomed cylinder restraints with a radius of 0.5 nm and a force constant of 10,000 kJ mol^−1^ nm^−2^ to keep the selected ion around the pore axis. We first pulled the selected ion from the bottom of the box across the channel to the top of the box. Then a series of frames were extracted from the pulling trajectory with a window spacing of 0.1 nm for the ion along the z direction. For each umbrella window, a 0.1-ns equilibration was performed, followed by 1-ns production simulations. The selected ion was restrained by harmonic potential with a force constant of 1,000 kJ mol^−1^ nm^−2^ along the z direction. The PMF analysis was performed by the weighted histogram analysis method in GROMACS (Kumar et al., 1992) with the cyclic reaction coordinate, and the standard deviations were estimated from 100 bootstraps. For TRPV6, we calculated the PMF of Ca^2+^ in the presence of a Ca^2+^/Na^+^ at the upper binding site. The Ca^2+^/Na^+^ was restrained around the upper binding site with a force constant of 10,000 kJ mol^−1^ nm^−2^. The selected ion was pulled from the lower solution region to the upper binding site and the window spacing was 0.05 nm for umbrella sampling.

### Data analysis

The pore radii of the channels were calculated using HOLE (Smart et al., 1996). The channel conductance of permeating ions with charge Q_ion_ was calculated based on the number of permeation events (N_Event_) over the entire trajectory. A permeation event was defined as one ion permeating the membrane through a channel from the extracellular side to the cytosolic side. The conductance (C) of a trajectory of a certain time length (t) under transmembrane potential (V_tm_) was calculated as follows:

$$C=\frac{I}{V_{tm}}=\frac{N_{Event}\times Q_{ion}}{t\times V_{tm}}.$$

The probability density of the ions in the pores on the R-z plane was calculated as follows:

$$\rho \left(R,z\right)=\frac{1}{2\pi N_{i}N_{f}}\sum_{i,f}\delta \left(R-R_{i,f}\right)\delta \left(z-z_{i,f}\right)/R_{i,f}\text{,}$$

where *R* represents the distance from the central axis of the channel, *z* is the coordinate perpendicular to the membrane, *N*_*i*_ the number of ions in the system, and *N*_*f*_ the number of frames recorded in the trajectory.

The coordination number of an ion is defined as the number of oxygen atoms in the first solvation shell of the ion. The radius of the first solvation shell used for both Na^+^ and Ca^2+^ is 3.0 Å in this study. In bulk solution, the coordination number is 7 for Ca^2+^ and 6 for Na^+^ in our simulations. The error of coordination number is the standard deviation of the mean values calculated from three independent trajectories, respectively.

To calculate the permeation transition probability among the binding sites in the SF, we monitored the trajectories of all the ions that came from the SF and left binding site S2 to enter the cavity. The number of ions that followed the permeation path S1→S2 was designated as N_s1_, the number of ions that followed the permeation path P→S2 was N_P_, the transition probability of S1→S2 was calculated as $\frac{N_{S1}}{N_{s1}+N_{P}}$, and the transition probability of P→S2 was $\frac{N_{P}}{N_{s1}+N_{P}}$.

### Online supplemental material

**Figure S1.**
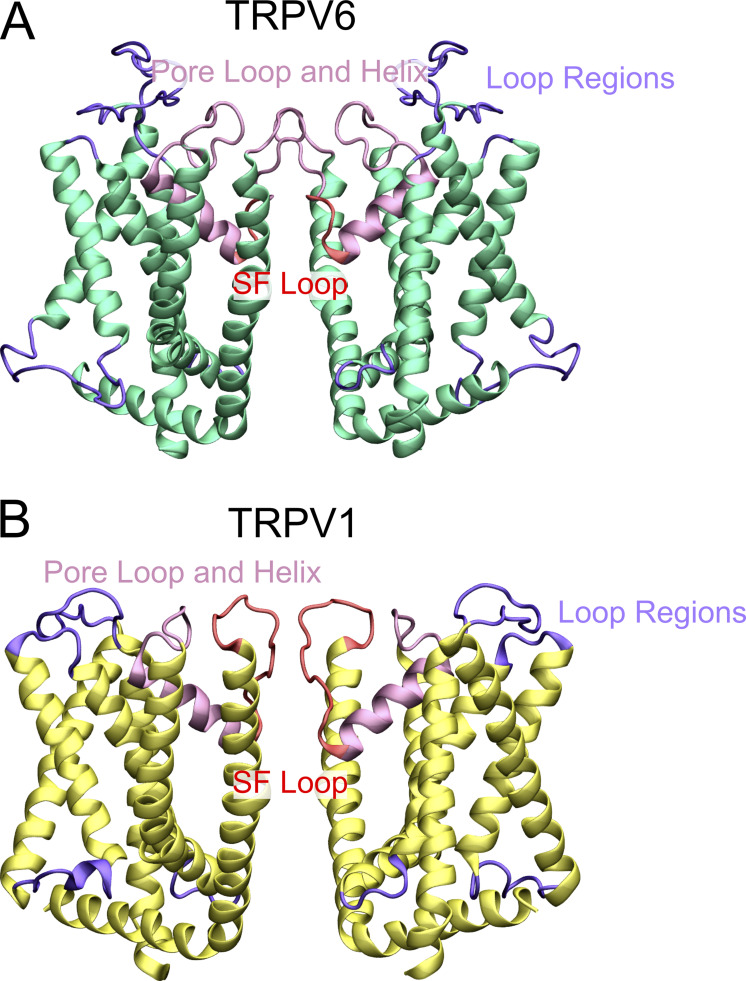
**Structural regions of transmembrane domains of TRPV6 and TRPV1. (A and B)** The transmembrane domain of TRPV6 (A) and TRPV1 (B) are shown as a cartoon with the SF loop colored red, the pore loop and helix other than SF loop colored pink, and all the loops other than the pore loop colored purple.

**Figure S2.**
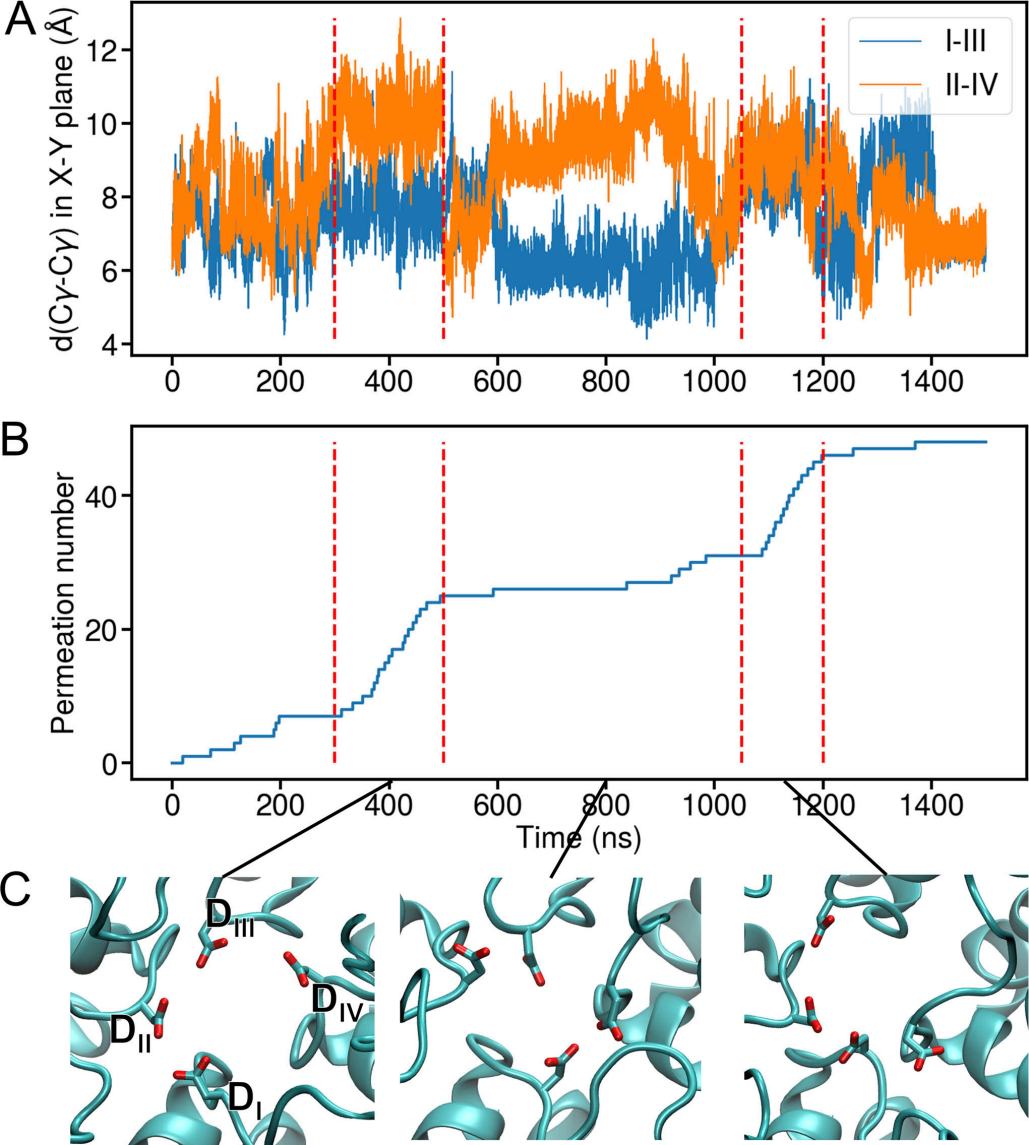
**Analysis of SF conformation at different conductance states of TRPV6. (A)** Time evolution of diagonal Cγ–Cγ distance in the x-y plane for DDDD (residue 542) locus. The three replicas are concatenated to be shown as one trajectory. The blue line indicates the Cγ–Cγ distance in the x-y plane for DI and DIII, while the orange line indicates the Cγ–Cγ distance in the x-y plane for DII and DIV. The red dashed lines indicate the high conductance states. **(B)** Time evolution of Ca^2+^ permeation number. The red dashed lines indicate the high conductance states. **(C)** Top view of three typical SF conformations extracted at 400, 800, and 1,100 ns, with D542 shown as sticks.

**Figure S3.**
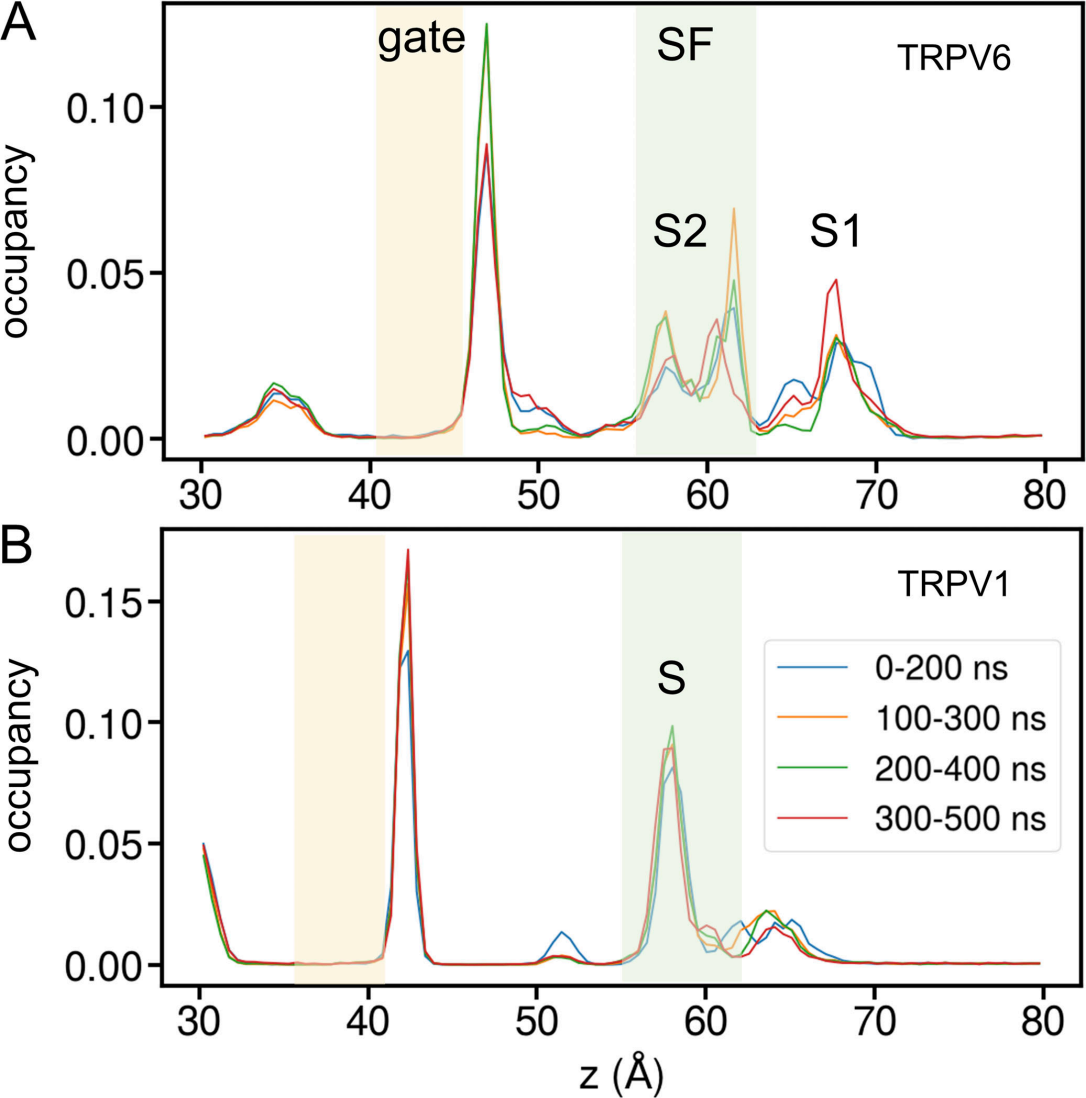
**Ion density convergence analysis. (A)** The convergence of Ca^2+^ density along the pore of TRPV6. Ca^2+^ around 3 Å of the pore axis were selected for analysis. The blue, orange, green, and red lines indicate the distribution estimated from 0–200, 100–300, 200–400, and 300–500 ns of three replicas, respectively. The intracellular gate and SF regions are indicated by orange and green shades, respectively. **(B)** Similar to A but for TRPV1. As can be seen, the ion distribution did not show a significant and systematic difference in these 200-ns time domains, indicating a quick convergence of ion distribution along the pore.

**Figure S4.**
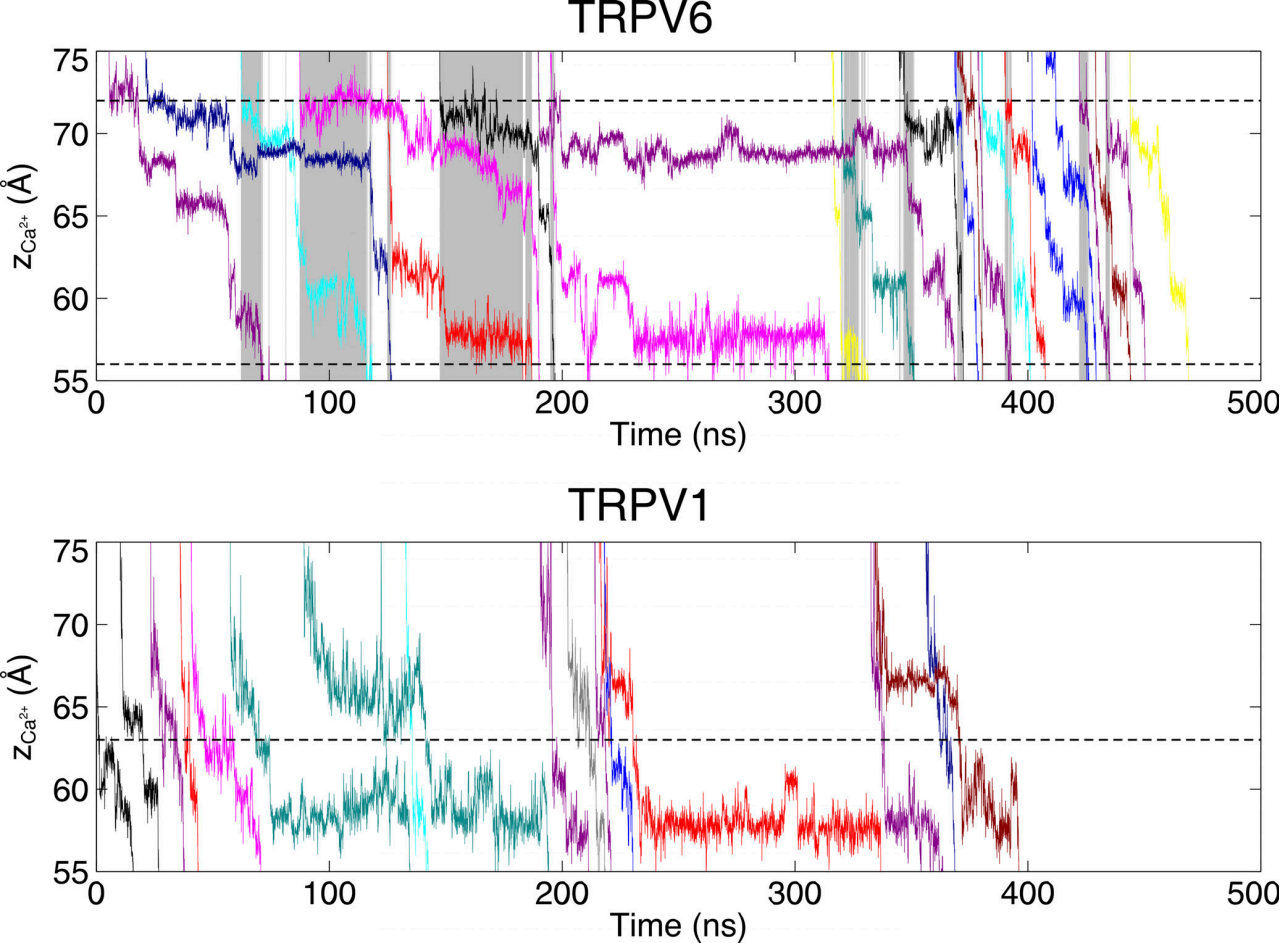
**The evolution of the z coordinates of permeating Ca**^**2+**^ **in the selectivity filter of TRPV channels.** This is the full 500-ns trajectory of Fig. 4. Different permeating cations are shown in different colors. Shaded areas indicate the simultaneous three-cation-occupy in the SF region. Dashed lines indicated the boundaries of the narrow pore region.

**Figure S5.**
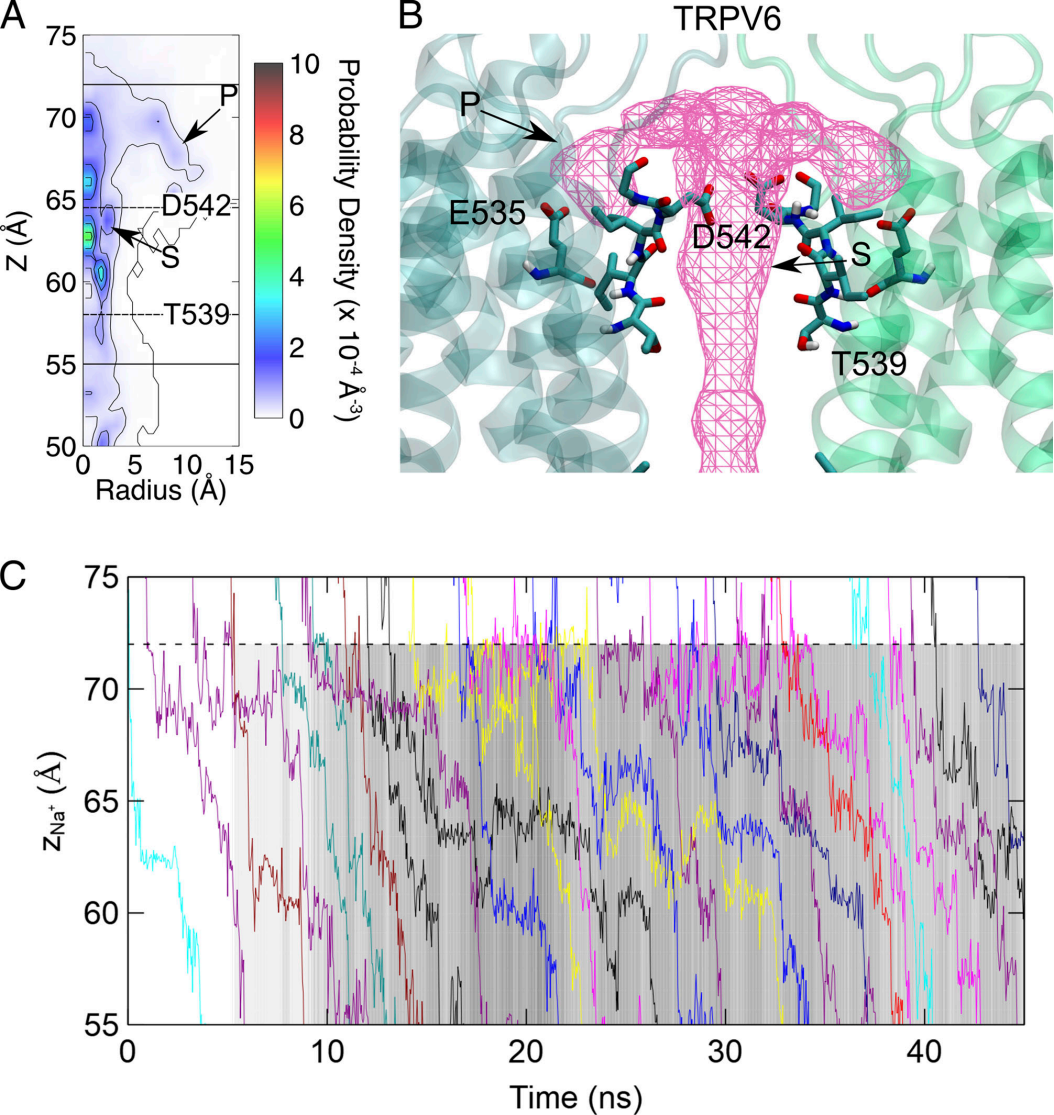
**Na**^**+**^ **binding and permeation pattern in the pore domain of TRPV6. (A)** The contour plot of Na^+^ density on the R-z plane around TRPV6. **(B)** Side view of the three-dimensional Na^+^ density map. Only two subunits are shown for clarity. The ion density is shown as the magenta isosurface. The binding sites and key residues are labeled. The density threshold used to plot the sodium isosurface is 0.1 Å^−3^. **(C)** Evolution of the z coordinates of permeating Na^+^ through the TRPV6 channel. The boundaries of the narrow pore region are indicated by dashed lines. The z coordinates of each Na^+^ are shown in different colors. The grayscale of the narrow pore region indicates the amount of Na^+^ occupying the region, with darker colors indicating a larger amount of Na^+^.

**Figure S6.**
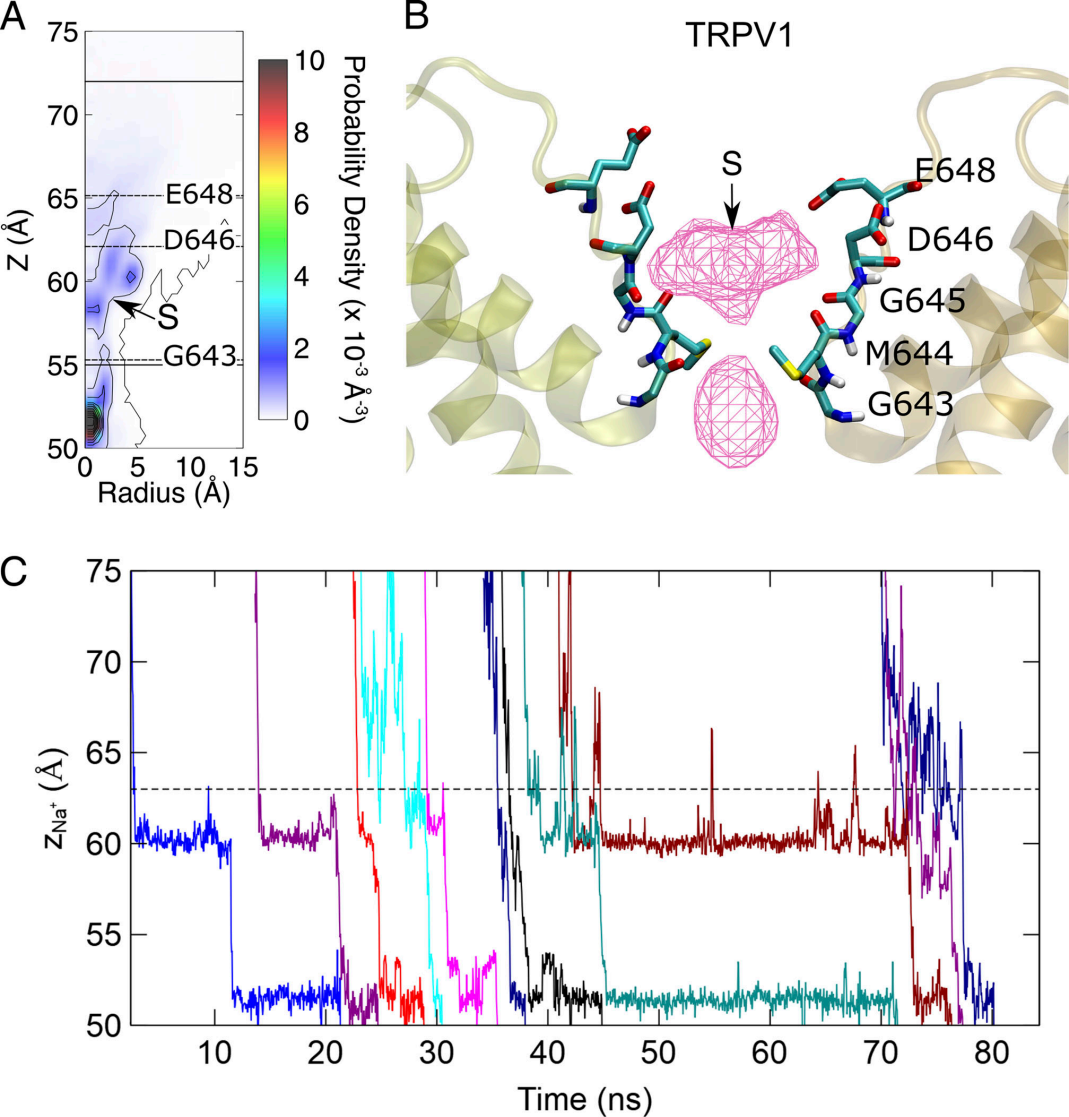
**Na^+ ^binding and permeation pattern in the pore domain of TRPV1. (A)** The contour plot of Na^+^ density on the R-z plane around TRPV1. **(B)** Side view of the three-dimensional Na^+^ density map. Only two subunits are shown for clarity. The ion density is shown as the magenta isosurface. The binding sites and key residues are labeled. The density threshold used to plot the sodium isosurface is 0.1 Å^−3^. **(C)** Evolution of the z coordinates of permeating Na^+^ through the TRPV1 channel. The boundaries of the narrow pore region are indicated by dashed lines. The z coordinates of each Na^+^ are shown in different colors.

Fig. S1 shows the structural regions of transmembrane domains of TRPV6 and TRPV1. Fig. S2 shows the analysis of SF conformation at different conductance states of TRPV6. Fig. S3 shows the ion density convergence analysis. Fig. S4 shows the evolution of the z coordinates of permeating Ca^2+^ in the SF of TRPV channels. Fig. S5 shows the Na^+^ binding and permeation pattern in the pore domain of TRPV6. Fig. S6 shows the Na^+^ binding and permeation pattern in the pore domain of TRPV1. Table S1 shows the equilibration protocol and parameters generated by CHARMM-GUI. Table S2 shows the restraint conditions that were tested in our MD simulations.

## Results

### The structure and flexibility of the pore helix influence the cation permeation of TRPV channels

**Figure 1.**
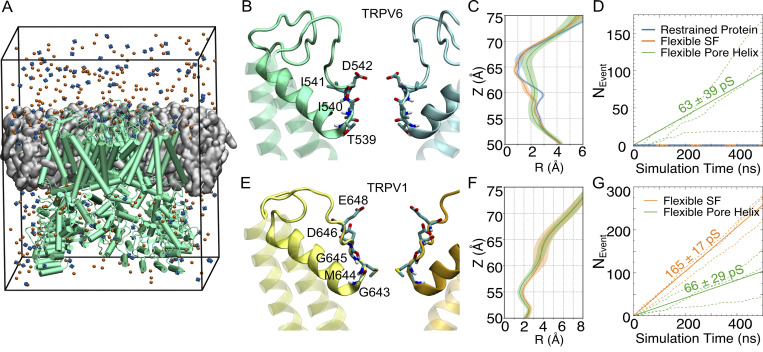
**The flexibility of the pore helix influences the cation permeation of TRPV channels. (A)** A representative simulation system, which contains the full TRPV6 (green cartoon) embedded in a POPC bilayer (gray surface). The multisite Ca^2+^ ions are shown as blue spheres and Cl^−^ as orange spheres. Water molecules are filled in the box but not shown for clarity. **(B)** The structure of the SF region of TRPV6. The backbone of protein is shown as a cartoon with the pore helix and loops shown as solid and other parts as transparent. Key residues forming the SF are shown as licorice and labeled. Only two chains are shown for clarity. **(C)** The average pore radius of the SF region of TRPV6 during simulations with different restraints. The standard deviation is shown as the shaded area. **(D)** The cumulative number of Na^+^ permeation events (*N*_*Event*_) during simulations with different restraints. The ion conductances calculated based on the number of permeation events were labeled. **(E–G)** Similar to B–D, respectively, but for TRPV1. The solid lines correspond to the average conductance from three independent simulation trajectories represented by dash lines.

In our atomistic simulations of the TRPV systems (Fig. 1 A), we applied position restraints to maintain the open conformation of the TRPV channels and found that various position restraints led to distinct permeabilities, particularly those restraints on the SF (Fig. 1 B and Fig. S1). In three 500-ns simulations of the pore domain of TRPV6, when the entire pore domain was restrained to the open-state cryo-EM structure (termed “restrained protein”), the narrow and rigid SF, with the entrance region formed by D542 having a radius of <1 Å (Fig. 1 C), was not permeable to cations and no single Na^+^ permeation was observed (Fig. 1 D). Removal of the restraints on the SF loop (termed “flexible SF”) gave limited flexibility to the SF and the radius was not significantly dilated (Fig. 1 C). No Na^+^ permeation was observed in three 500-ns simulations under such conditions for TRPV6 (Fig. 1 D). To further relax the SF region, the loops and pore helix between S5 and S6 were made fully flexible (termed “flexible pore helix”). Under such a condition, the continuous permeation of Na^+^ was observed (Fig. 1 D). The radius of the pore was dilated to ∼1.5 Å at the SF and the fluctuation increased compared with that in the more restrained situations (Fig. 1 C), allowing the passage of sodium through the SF (Fig. 1 D). The conductance calculated from the simulation with the flexible pore helix (∼62 pS) is comparable with the experimental value of 42–58 pS (Yue et al., 2001). These results highlight the importance of SF flexibility for ion permeation.

In TRPV1, the flexibility of the pore loops and helix (Fig. 1 E) also influenced the radius at SF during the simulations, thus impacting cation permeation. The open-state structure used in this study was solved using the spider toxin peptide DkTx and the vanilloid agonist RTX, whose binding to TRPV1 induces pain in humans. DkTx binds to the extracellular part of the channel and induces a conformational change in the loop between S5 and S6 (Gao et al., 2016). Contrary to TRPV6, the less restrained SF of TRPV1 showed a slightly smaller radius (Fig. 1 F), whereas the number of permeation events dropped by more than half (Fig. 1 G), resulting in a conductance of ∼66 pS, close to the experimental value (62.8 pS; Samways and Egan, 2011).

Simulations of TRPV6 and TRPV1 with different restraints applied to the pore loops and pore helix revealed that the flexibility of this region is essential for cation permeation, which is probably due to the resulting differentiable pore radii when the SF adopts permeating ions (Fig. 1, C and F). Based on the above benchmarking, position restraints were only applied to the transmembrane helices in the following production simulations, leaving the loop region and pore helix between the transmembrane helices fully flexible. It should also be noted that our simulations were conducted under a high voltage of 500 mV, so the resulting conductance may be overestimated when compared with electrophysiological measurements under physiological conditions if the I-V curves were non-linear. This was also observed in previous studies on potassium channels, and it was encouraging to see that the ion binding and permeation mechanisms were preserved (Jensen et al., 2013).

**Figure 2.**
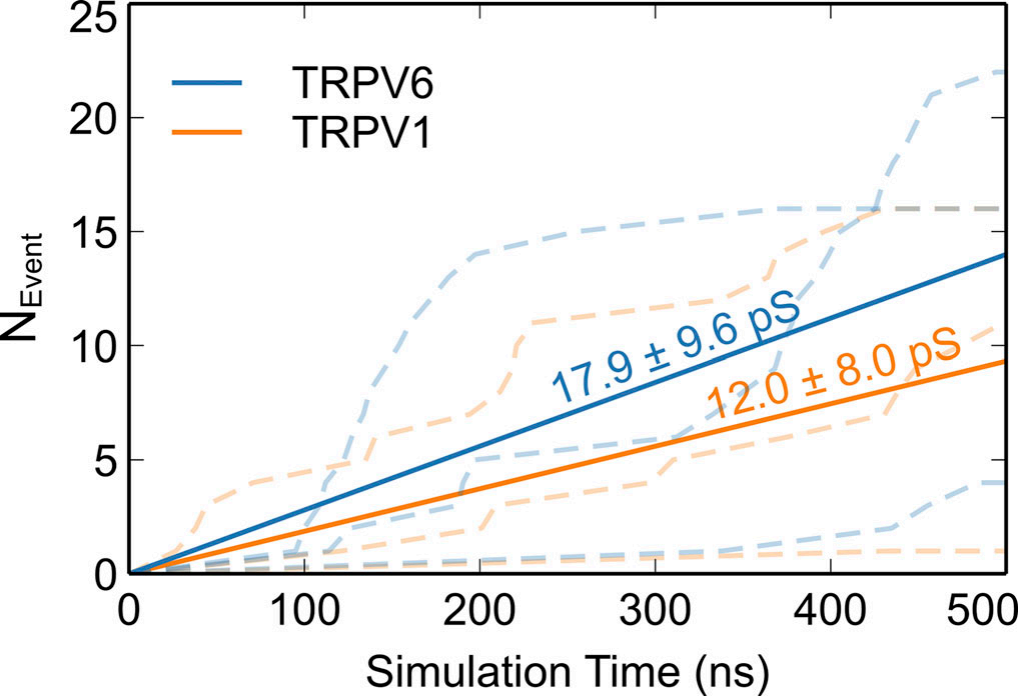
**Ca**^**2+**^ **permeation through TRPV6 and TRPV1.** The dashed lines illustrate the cumulative number of Ca^2+^ permeation events (N_Event_) during simulations. The ion conductance was calculated based on the number of permeation events from three independent simulations.

Under these conditions, we observed continuous Ca^2+^ permeation events in both channels and measured Ca^2+^ conductance as ∼18 pS for TRPV6 and 12 pS for TRPV1 (Fig. 2). These are close to experimental single-channel conductance of TRPV channels, which are summarized in Table 1. It is also noticeable that both Na^+^ and Ca^2+^ showed burst-like permeation patterns (Figs. 1 and 2), which is more obvious for Ca^2+^ than Na^+^. When breaking down the concatenated trajectory, we observed that the high-permeability burst corresponds to a more dilated SF than the low-permeability trajectory and the side-chain conformational change of D542 is directly involved in the ion permeation through TRPV6 (Fig. S2). This suggests that the ion permeation is highly sensitive to the specific SF conformation.

**Table 1.** The overall conductance in simulations with different concentrations of cations

Ion channel	Calcium (150 mM)	Sodium (150 mM)	Sodium + calcium (150 mM + 150 mM)	
	Conductance (pS)		Conductance (pS)	$N_{Event}^{Ca^{2+}}:N_{Event}^{Na^{+}}$
TRPV6	17.9 ± 9.6	141 ± 25 (42–58^a^)	7.4 ± 4.6	2.1
TRPV1	12.0 ± 8.0 (15.2^b^)	66 ± 29 (62.8^b^)	27 ± 16	7.4

The experimental conductance is shown in the brackets.

a Yue et al. (2001).

b Samways and Egan (2011).

### Differences in Ca^2+^ binding sites and knock-off permeation of TRPV1 and TRPV6

**Figure 3.**
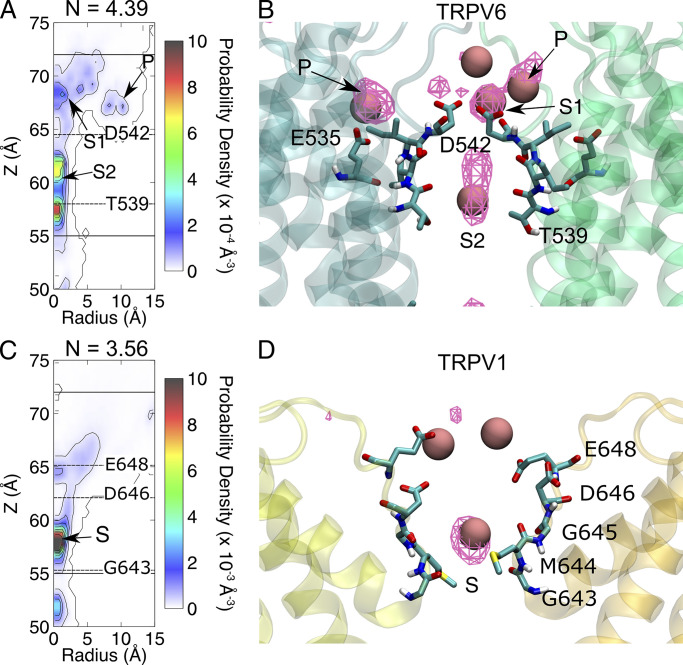
**The difference in Ca**^**2+**^ **binding at the SF of TRPV channels leads to different ion permeation patterns. (A)** The contour plot of Ca^2+^ density on the R-z plane of the SF region of TRPV6. Binding sites are indicated by arrows and labeled. The Cα positions of key residues are indicated by dashed lines. The boundaries of the SF are indicated with solid lines. N is the average number of Ca^2+^ in the SF region. **(B)** The representative snapshot of Ca^2+^ binding at the SF region. The average ion density map is shown as a meshed isosurface. Binding sites are indicated by arrows and labeled. Residues forming the binding sites are shown as licorice. The Ca^2+^ is shown as a pink sphere. The backbone of protein is shown as a transparent cartoon. Only two chains are shown for clarity. The ion density isosurface is plotted using a threshold of 0.1 Å^−3^. **(C and D)** Similar to A and B, respectively, but for TRPV1.

Our MD simulations showed that the Ca^2+^ binding within the pore achieved convergence within 200 ns (Fig. S3) and that the binding sites of Ca^2+^ in the SF regions of TRPV6 and TRPV1 were distinct. In TRPV6, two types of Ca^2+^ binding sites were identified: site S at the center of the SF and the peripheral binding site P (Fig. 3, A and B). Binding site S can be divided into two subsites: S1 and S2. S1 is located at the entrance of the SF and Ca^2+^ interacts with the D542 side chain, which was occupied 52% of the simulation time. S2 is located in the lower SF, which is a rather extended binding site that can be occupied by only one Ca^2+^ at a time. Ca^2+^ mainly interacts with the carboxyl group of T539 at S2, which was occupied 73% of the simulation time. Ca^2+^ at the binding site P mainly interacts with the negatively charged residue E535. Covering the above binding sites (both S and P), we defined the SF region of TRPV6 as the region formed by residues T539–D542. Notably, the binding sites observed in our simulations were not identical to the ion densities identified in previous structural studies. Compared with the closed-state crystal rTRPV6 structure (Saotome et al., 2016), our simulations showed two binding sites at the SF with higher electron density in the crystal structure, but the third binding site with lower electron density in the cavity was not obvious in our simulations. The open-state cryo-EM hTRPV6 structure solved the binding site located in the middle of the SF, but no Ca^2+^ density was observed at the entrance of the SF (McGoldrick et al., 2018). These differences between our simulations and existing structures can be partly explained by the gating state and flexibility of SF during ion permeation. The crystal structure was in a closed state, whereas it was difficult to capture the less stable ion binding sites in the cryoEM structure; therefore, our simulations provide more details on the ion distribution while permeating. In addition, the transmembrane potential applied in our simulations may cause a shift in the ion binding site compared with the experimental observation under transmembrane potential-free conditions.

In TRPV1, only one stable Ca^2+^ binding site at SF was observed, which was located above the narrowest site formed by G643 (Fig. 3, C and D) and occupied 86% of the simulation time. Ca^2+^ bound to this site mainly interacts with G643, M644, and G645. No peripheral Ca^2+^ binding site corresponding to site P in TRPV6 was observed in TRPV1. To be comparable with TRPV6, we defined the TRPV1 SF region in a similar way to that of TRPV6, including the wide extracellular vestibule and the narrowest site at G643.

**Figure 4.**
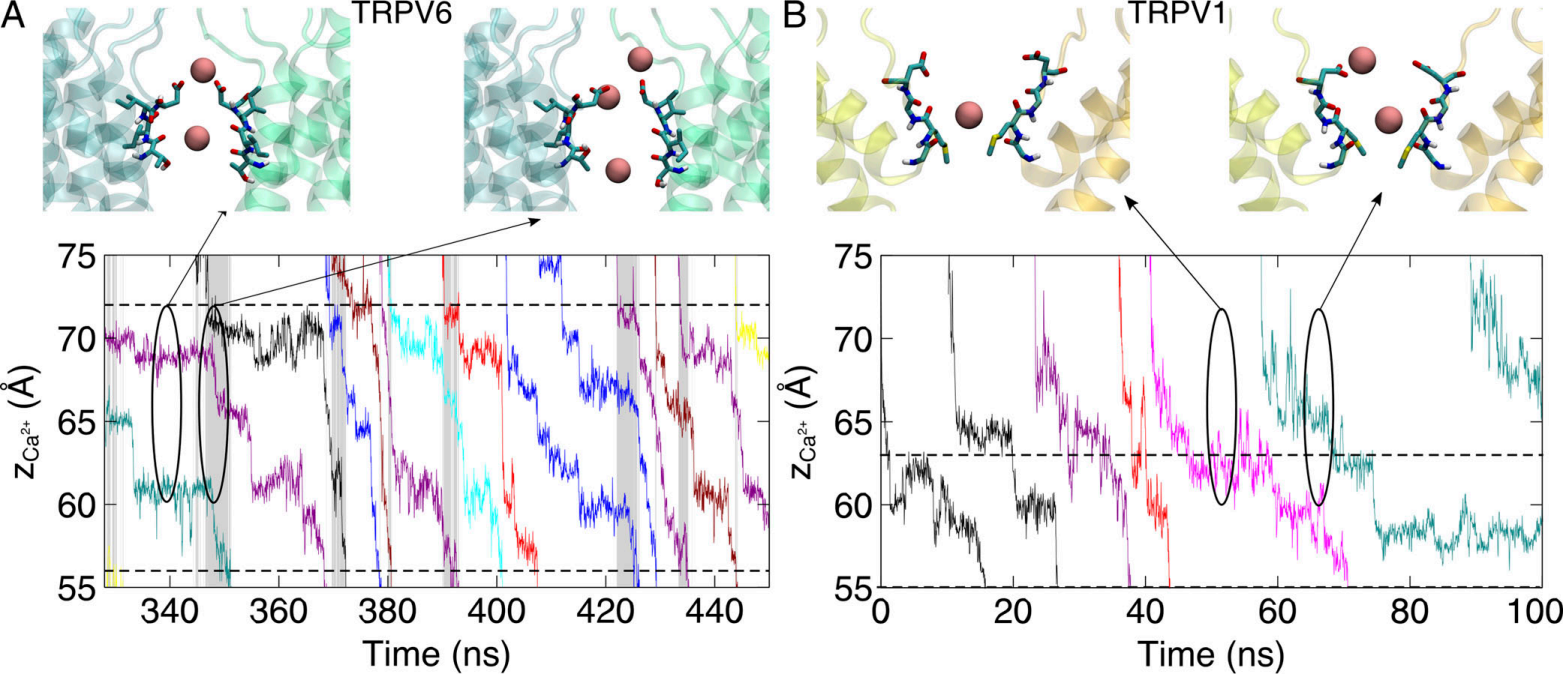
**Different modes of Ca**^**2+**^ **permeation in TRPV6 and TRPV1. (A)** Bottom: The evolution of the z coordinates of permeating Ca^2+^ in TRPV6. Different permeating Ca^2+^ are shown in different colors. Shaded areas indicate the simultaneous 3-Ca^2+^ occupancy in the SF region. Dashed lines denote the boundaries of the narrow pore region. Top: The representative snapshot of Ca^2+^ permeating the SF region. Residues forming the SF are shown as licorice. The Ca^2+^ is shown as a pink sphere. The backbone of protein is shown as a transparent cartoon. Only two chains are shown for clarity. **(B)** Similar to A, but for TRPV1.

Different Ca^2+^ binding sites in the SF region led to different modes of permeation of TRPV6 and TRPV1. In both TRPV channels, a Ca^2+^ transport process consistent with a previously proposed knock-off mechanism was observed (Tsien et al., 1987). Continuous permeation of Ca^2+^ requires the entry of Ca^2+^ into the SF region to knock off Ca^2+^ at the binding site. However, in TRPV6, since two Ca^2+^ ions are bound at the SF, permeation is characterized by a third incoming Ca^2+^, sequentially knocking the two bound Ca^2+^ in the SF. As shown in Fig. 4 A and Fig. S4, when an incoming Ca^2+^ approaches the binding site S1 of TRPV6 from the extracellular site, it knocks the Ca^2+^ bound at S1 by electrostatic repulsion. Sequentially, the Ca^2+^ bound at S1 knocks the nearby Ca^2+^ bound at S2, leading to the dissociation of Ca^2+^ to enter the channel cavity for permeation. Meanwhile, the Ca^2+^ bound at S1 moves to S2 and then S1 is occupied by a newly entering Ca^2+^ from the extracellular site. The three Ca^2+^ ions move in concerted and synergic ways to permeate, while the binding site P is not directly involved in the permeation. In contrast, for TRPV1, the calcium ion permeation process involves only one bound Ca^2+^ within the SF and one incoming Ca^2+^ from the extracellular side, without sequential knocking or concerted movement between multiple ions bound in the SF (Fig. 4 B and Fig. S4).

The collective ion permeation was also observed for Na^+^ in TRPV6 and TRPV1 in the single-cation simulations. At maximum, four and two Na^+^ can occupy the SF of TRPV6 and TRPV1, respectively (Figs. S5 and S6). A more continuous and larger ion density was observed for Na^+^ than for Ca^2+^ within both channels, partially explaining the more efficient Na^+^ permeation than Ca^2+^ in the single-cation simulations. It should be noted that here we only focus on the ions in the SF, while the ions in the cavity would probably be involved in the concerted permeation as well, as observed in the work by Ives et al. (2023). Therefore, multiple ion binding and concerted permeation appear to be a universal phenomenon in the TRPV channels.

### The desolvation of Ca^2+^ is slightly different in TRPV6 SF than in TRPV1

**Figure 5.**
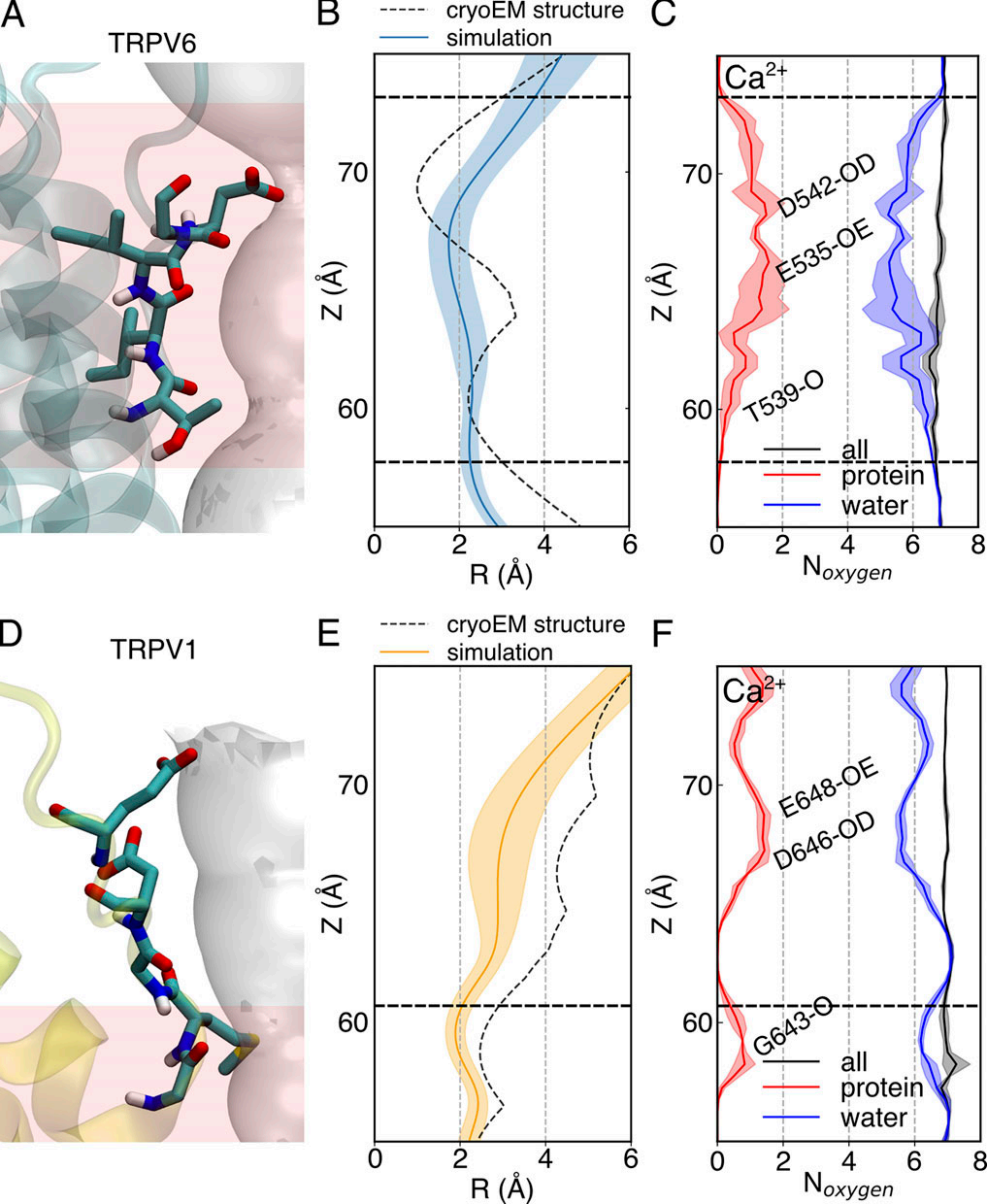
**The dehydration of calcium through the SF of TRPV6 and TRPV1. (A)** The residues forming the SF of TRPV6. **(B)** The SF pore radius along the pore axis of the open-state TRPV6. The dashed curve is calculated from the cryo-EM structure. The solid line is the average pore radius from MD simulations, with the shaded area representing the standard error of the mean. **(C)** The total number (black) of coordinated oxygen atoms around the Ca^2+^ within the pore, and the contributions from protein (red) and water (blue), respectively. The significant contributions from protein oxygen are marked with corresponding protein residue IDs containing the oxygen (O: oxygen atom in the backbone; OD: oxygen atom at the side chain of aspartic acid; OE: oxygen atom at the side chain of glutamic acid). The solid lines represent the average values and the shaded areas represent the standard error of the mean from three independent simulators. The dashed straight lines indicate the boundaries of narrow pores with a radius <3 Å in the cryo-EM structure. **(D–F)** Similar to A–C, respectively, but for TRPV1.

The dehydration of permeating Ca^2+^ in the SF region has similarities but also shows discrepancies between TRPV6 and TRPV1 (Fig. 5). Ca^2+^ desolvation occurs within the SF region of both TRPV6 and TRPV1, but the desolvation in TRPV6 occurs in a longer region than that observed in TRPV1 (Fig. 5, A and B). In TRPV6, at most 1.5 water molecules in the first solvation shell were removed, and this dehydration was mainly replaced by oxygen atoms on the side chains of charged residues (D542 and E535) in the SF region (Fig. 5 A). In TRPV1, desolvation of permeating Ca^2+^ at the SF in the TRPV1 channel occurred only at the narrowest point formed by G643, where Ca^2+^ was coordinated by the oxygen atoms of the protein backbone (Fig. 5 B). It should be noted that although partial dehydration of Ca^2+^ occurs at negatively charged residues E648 and D646 above the SF of TRPV1, the pore radius at this location is larger than the radius of the first solvation shell formed by coordinated waters around Ca^2+^ (∼4 Å). Therefore, we consider the desolvation of the calcium ion here not due to spatial constraints but due to the stronger electrostatic attraction between the Ca^2+^ and the negatively charged residues. In addition, the calcium ions here do not necessarily participate in the permeation, so the ion desolvation here was not considered as permeation dehydration in this study. Therefore, the dehydration of permeant Ca^2+^ mainly occurs around the upper SF of TRPV6, whereas in TRPV1, this mainly occurs around the constriction site in the lower SF. When dehydration occurs at these constriction sites, there are always oxygen atoms of proteins that can replace water oxygens to coordinate with the permeant Ca^2+^ (Fig. 5).

### The blockage effect of Ca^2+^ on monovalent current and valence selectivity

**Figure 6.**
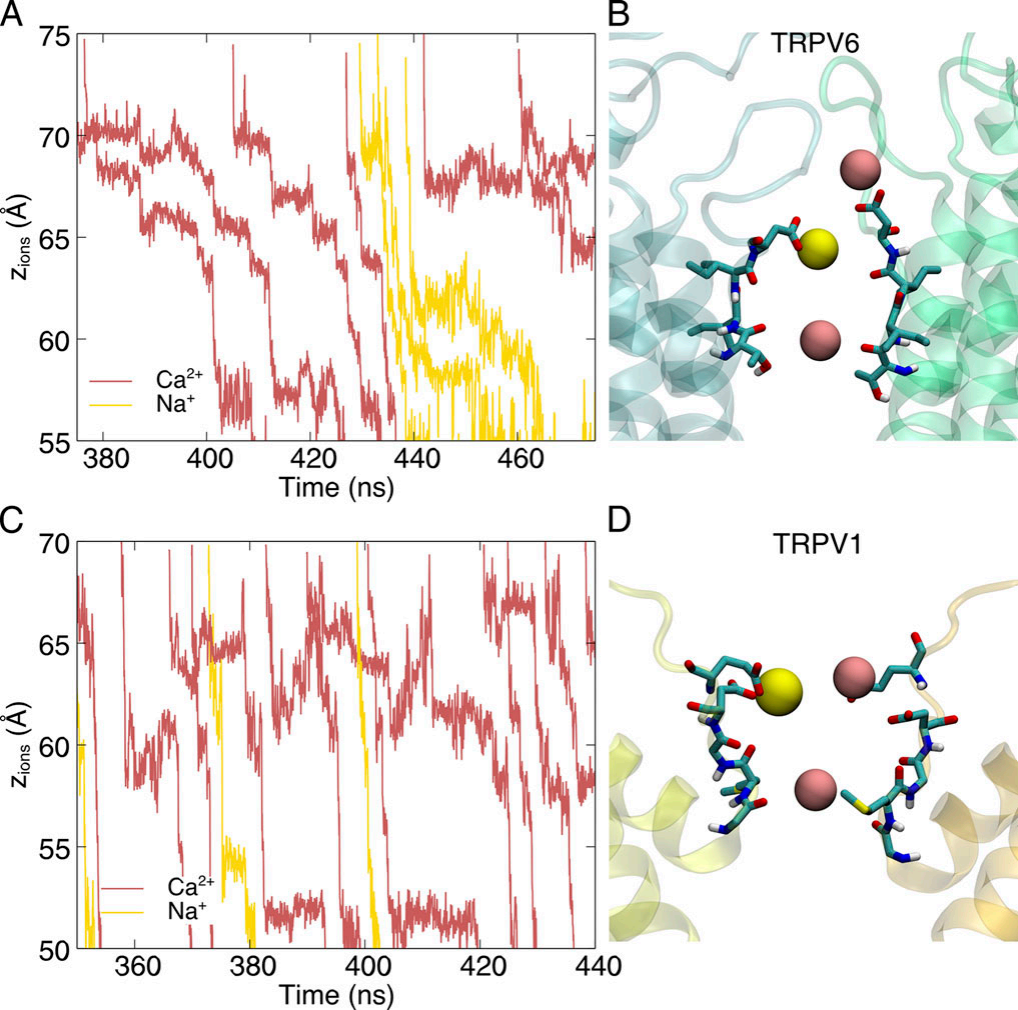
**Na**^**+**^ **shows different permeation behavior in TRPV6 and TRPV1 in the presence of Ca**^**2+**^**. (A)** Evolution of the z coordinates of TRPV6-permeating cations in the dicationic simulations. **(B)** Representative conformation during Na^+^ permeation of TRPV6. The backbone of protein is shown as a transparent cartoon. Residues forming the SF region are shown as licorice. Only two chains are shown for clarity. Ca^2+^ is shown as the pink sphere and Na^+^ as the yellow sphere. **(C and D)** C is similar to A and D is similar to B, but for TRPV1.

In our simulations with high-concentration dicationic solutions (150 mM Ca^2+^ and 150 mM Na^+^), the blockage effect of divalent ions on monovalent currents was observed for both TRPV6 and TRPV1, although Na^+^ permeation was not fully precluded. The conductance of the channel decreased significantly when Ca^2+^ was added to the Na^+^ solution (Table 1). The stronger blockage effect observed in simulations with TRPV6 than with TRPV1 (19.1-fold decrease in TRPV6 versus 2.5-fold decrease in TRPV1) is consistent with experimental observations (Vennekens et al., 2000; Samways and Egan, 2011), which may be related to the stronger Ca^2+^ selectivity of TRPV6 over TRPV1 (Gillespie and Boda, 2008; Table 1). Despite the blockage effect of Ca^2+^, permeation events of Na^+^ were still observed in our simulations. The Na^+^ permeation patterns differed between TRPV6 and TRPV1. In TRPV6, Na^+^ is not permeable when Ca^2+^ binds to the SF. Only at the time interval between Ca^2+^ permeation when one Ca^2+^ leaves the binding site S1 can the nearby Na^+^ take the opportunity to bind to this site and cut in line to permeate (Fig. 6, A and B). Since the binding Ca^2+^ moves in concert during permeation, another Ca^2+^ will occupy the binding site soon after one Ca^2+^ leaves, so the time interval available for Na^+^ to cut in line is rather limited. Thus, in TRPV6, calcium ions show a strong blocking effect on monovalent currents. In contrast, the vestibule above the SF region in TRPV1 is rather spacious; when one Ca^2+^ is present in this region, Na^+^ can bypass it and permeate (Fig. 6, C and D). This bypass permeation pattern of Na^+^ in TRPV1 is more likely to occur than the cut-in-line pattern in TRPV6. Therefore, the blockage effect of Ca^2+^ on monovalent currents is weaker in TRPV1 than in TRPV6.

In the high-concentration dicationic systems, TRPV6 and TRPV1 showed slight valence selectivity in our MD simulations. As shown in Table 1, TRPV1 showed a slight preference for Ca^2+^ permeation over Na^+^ (∼7:1), which is consistent with the previous conclusion that TRPV1 is not Ca^2+^ selective. However, for TRPV6, we did not observe any significant Ca^2+^ selectivity either. The ratio of permeation events was ∼2:1 (N_Ca_:N_Na_) in our simulations with 150 mM Ca^2+^ and 150 mM Na^+^, which is inconsistent with the prediction that the permeability ratio P_Ca_:P_Na_ is > 100:1 under physiological conditions. This is discussed further in the next section.

### Binding site competition at SF entrance facilitates Ca^2+^ selectivity/Na^+^ blockage in TRPV6

**Figure 7.**
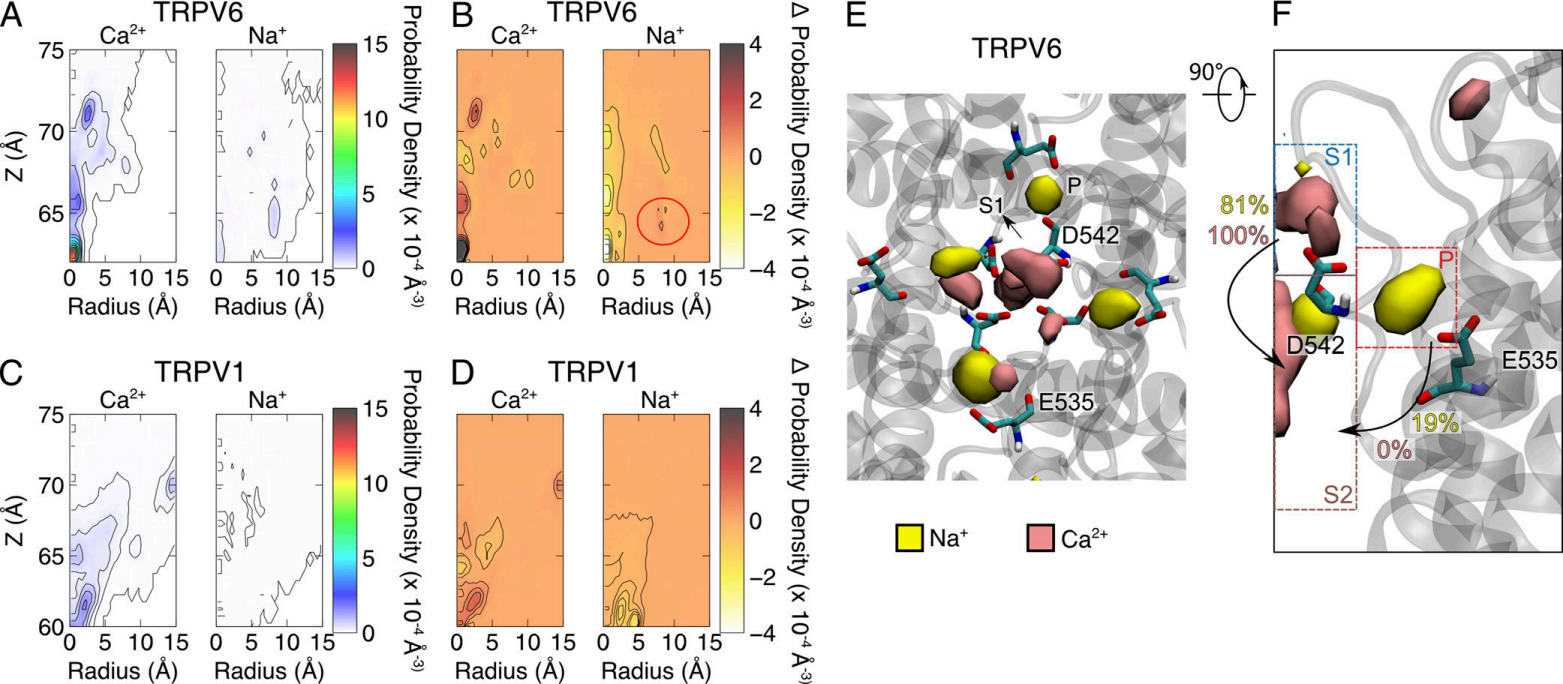
**The binding site differentiation between Ca**^**2+**^ **and Na**^**+**^ **in the SF region of TRPV6 in dicationic solution. (A)** The contour plot of cation density on the R-z plane of the SF region of TRPV6. **(B)** The difference in cation density calculated by subtracting the density of the one-cation-species system from the density of the dicationic system. The peripheral (P) binding site is indicated by the red circle. **(C and D)** C is similar to A and D is similar to B, but for TRPV1. **(E)** Top view of the density map of cations in the SF region of TRPV6. The backbone of the protein is shown as the gray transparent cartoon. The density map is shown as the solid isosurface. The binding sites and key residues are labeled. Only two chains are shown for clarity. **(F)** Side view of E. Only one chain is shown. Binding sites are indicated by dashed rectangles. The transition probabilities of S1→S2 and P→S2 are labeled on the corresponding arrows, with yellow for Na^+^ and pink for Ca^2+^. The ion density isosurface is plotted using a threshold of 0.1 Å^−3^.

In the vestibule above the SF, Ca^2+^ and Na^+^ compete to occupy binding site S1. In our dicationic simulations, a significant decrease in the density of Na^+^ in the SF region of both TRPV6 and TRPV1 was observed compared to the system with only Na^+^ as a cation, indicating that Ca^2+^ repels Na^+^ from this region (Fig. 7, A and C). In TRPV1, Na^+^ density in the entire SF region decreased (Fig. 7 D). In contrast, in TRPV6, Na^+^ density showed a slight increase at the peripheral binding site P (red circle in Fig. 7 B). The differentiation of Ca^2+^ and Na^+^ binding in TRPV6 SF was observed by a detailed inspection of their 3D densities. Ca^2+^ mainly binds to the central binding site S, including S1 and S2, whereas Na^+^ is preferentially bound at the peripheral binding site P (Fig. 7 E). Ion transition probability analysis between these binding sites showed that for both Ca^2+^ and Na^+^, the ions bound at S2, which is the indispensable site during permeation, were all transferred from the S1 binding site in the single cationic systems, indicating that S1→S2 is the major permeation path. In the dicationic systems, all of the Ca^2+^ ions still followed this single permeation path, while for Na^+^, 81% of the permeation events followed the S1→S2 path and 19% followed a new P→S2 path. Most of the permeating ions once bound at site P moved to the S1 binding site for further permeation to S2, but some of them still moved directly from the binding site P to S2 (Fig. 7 F). This inequality in the transition rate to S2 indicates that the ions bound at site S1 have a significantly higher permeation probability, and those bound at site P are less involved in direct permeation. In the TRPV6 dicationic simulation system, Ca^2+^ dominantly occupied the central binding sites, having a higher probability of permeation, while Na^+^ was expelled to the peripheral binding sites P, having a lower probability of permeation. This binding site competition and differentiation between cations of various valences may also contribute to selectivity for Ca^2+^ or blockage of Na^+^ in TRPV6.

### Potential of mean force for Na^+^ and Ca^2+^ permeation through TRPV1 and TRPV6

**Figure 8.**
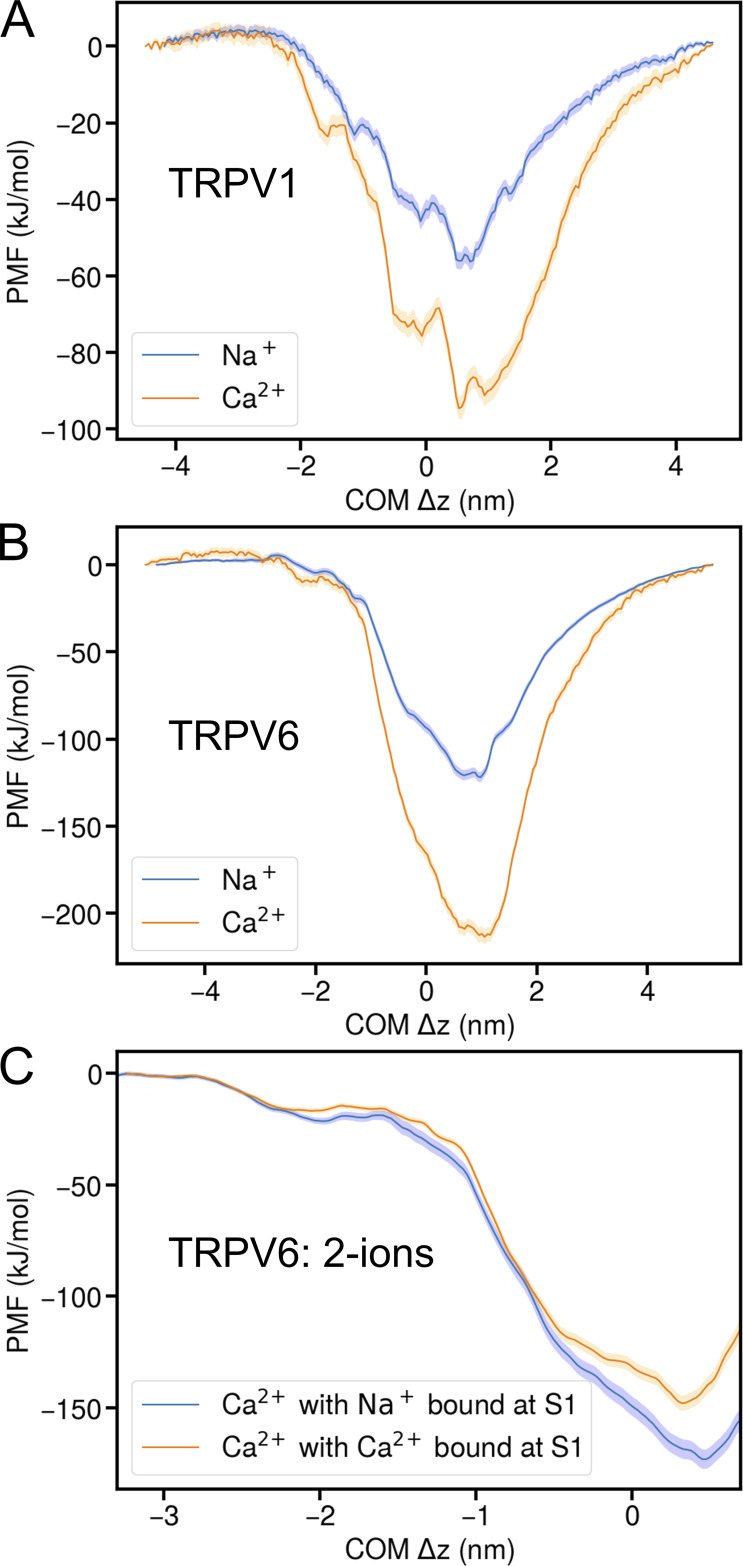
**PMF of Ca**^**2+**^**/Na**^**+**^ **in TRPV1 and TRPV6. (A)** PMF of Ca^2+^/Na^+^ in TRPV1. The center-of-mass deviation (COM Δz) measures the z distance between the ion and the COM of the protein. The blue solid line indicates the PMF for Na^+^ while the orange solid line is for Ca^2+^. The shades indicate the standard deviation estimated from bootstrap calculations. **(B)** Similar to A but for TRPV6. **(C)** PMF of Ca^2+^ in TRPV6 with an additional ion bound at the S1 binding site. The blue solid line indicates the PMF for Ca^2+^ with a Na^+^ bound at S1, while the orange solid line indicates the PMF for Ca^2+^ with a Ca^2+^ bound at S1. The shades indicate the standard deviation estimated from bootstrap calculations.

To gain quantitative insights on the ion binding competition at the SF, we conducted PMF calculations for both TRPV1 and TRPV6. As shown in the previous section, multiple cations were not only bound at the vestibule or SF but were also directly involved in the collective ion permeation. Calculating the PMF of multiple-ion permeation is very challenging as the calculation is multidimensional and hard to converge. Therefore, we adopted a simplified albeit non-physiological strategy to calculate single-ion PMFs. We removed all the surrounding ions from the protein and calculated single-ion PMFs while one ion translocated through the channels. As shown in Fig. 8, A and B, both TRPV1 and TRPV6 exhibit a strong cation binding affinity at the SF, with a strong preference toward Ca^2+^. For TRPV1, the binding strength to Na^+^ and Ca^2+^ are about −56.3 and −94.6 kJ/mol at the SF. For TRPV6, these values are about −121.9 and −213.5 kJ/mol, respectively. Obviously, the SF of both channels tends to attract cations, and the attraction is much stronger in TRPV6. Such strong binding affinities indicate that it is highly unlikely for a single cation to permeate through the channels, and multiple cations must occupy the vestibule or SF to weaken the binding strength before any permeation can occur. As expected, the binding preference of Ca^2+^ over Na^+^ for TRPV6, as represented by ΔΔ*E*_*bind*_ = 91.6 kJ/mol, is significantly stronger than that for TRPV1 (ΔΔ*E*_*bind*_ = 38.3 kJ/mol). It should be noted that these are non-physiological conditions without any environmental ions around the protein, so the absolute binding strength from the PMF does not mean much. However, the binding strength difference (ΔΔ*E*_*bind*_) is still qualitatively informative, indicating that the SF of TRPV6 should have a stronger binding preference toward Ca^2+^ than TRPV1 under very low ion concentrations.

As TRPV6 shows a two-ion binding feature within the SF, we examined how the binding of an additional cation at the S1 site affects the PMF of the translocating ion that moves from the S2 site to the cytosolic side (Fig. 8 C). When an additional Ca^2+^ is bound at S1, the depth of the PMF well at the SF changed from −213.5 to −147.9 kJ/mol. This means that the binding of an additional Ca^2+^ at S1 reduced the binding strength of the translocating Ca^2+^ within the SF for about 65.6 kJ/mol. A similar effect was observed if a Na^+^ is bound at S1, but the binding strength of the permeating Ca^2+^ is only reduced for about 40.5 kJ/mol. These results suggest that an incoming cation that binds to S1 can facilitate the permeation of the Ca^2+^ that preoccupies S2 within the SF, and the effect is more significant if the incoming ion is Ca^2+^. Again, these PMF calculations were conducted under highly simplified conditions, so these two-ion PMFs do not correspond to the observed ion permeability in previous simulations or the physiological condition in the cell. The binding strength calculated this way is much stronger than expected from the experimentally observed ion conductance under physiological conditions. However, the difference of reduced binding strength is quantitatively informative if one takes the approximations that the environmental ion distribution was not affected by the S1-bound ion type and the interactions between atoms/ions are pairwise and additive. The difference value of ∼25 kJ/mol indicates that an S1-bound Ca^2+^ indeed exerts a much stronger driving force on the S2-bound Ca^2+^ than an S1-bound Na^+^ does.

## Discussion

Various Ca^2+^-permeable channels showed significantly different Ca^2+^ selectivity, with reported permeability ratios over monovalent cations ranging from ∼1:1 to ∼1,000:1, which are required for these channels to execute their respective biological functions (Sather and McCleskey, 2003). How ion selectivity of various degrees is achieved has long been an intriguing question in the field of ion channel research, which requires detailed structural and dynamic insights at the atomic level. Homologous ion channels that show distinct Ca^2+^ selectivity and whose structures have been resolved, such as the TRPV channels studied in this work, provide an excellent opportunity to address this issue.

Our MD simulations explored cation permeation in both Ca^2+^-selective TRPV6 and non-selective TRPV1 channels under non-physiological (high [Ca^2+^] and high voltage) conditions. Unfortunately, despite the higher Ca^2+^/Na^+^ concentration ratio used in simulations than physiological conditions, the experimentally observed ion selectivity could not be reproduced under the simulation conditions. Nevertheless, our simulations provided insights indicating that competition between cations for multiple Ca^2+^ binding sites within the SF is likely the primary step to discriminate between Ca^2+^ and Na^+^ ions during permeation. From the permeation trajectory and PMF analysis, it was observed that Ca^2+^ showed a much stronger binding preference than Na^+^ at the SF entrance of both TRPV6 and TRPV1. In the presence of Ca^2+^, Na^+^ ions were expelled from the central binding site (S1) at the SF entrance (Fig. 7). Although Na^+^ can still occupy the lateral binding site P near the SF entrance of TRPV6, the binding site P does not lead to efficient permeation into the SF. The preferential binding of Ca^2+^ at the SF entrance can generate a blocking effect on Na^+^, which can enhance the permeation probability of Ca^2+^ over Na^+^ for both TRPV6 and TRPV1.

The number of binding sites in SF can generate distinct permeation patterns. With one Ca^2+^ binding site in the SF, Na^+^ can still frequently find their chance to enter the SF, as observed in TRPV1. A possible factor that can enhance Ca^2+^ selectivity is the presence of multiple Ca^2+^ binding sites in the SF, as observed in the SF of TRPV6. When the lower Ca^2+^ binding site (S2) is occupied by Ca^2+^, the upper binding site (S1) can be occupied by either Ca^2+^ or Na^+^, with a stronger binding preference for Ca^2+^. Meanwhile, only when S1 is occupied by Ca^2+^ does the Ca^2+^ at the S2 site in the SF receive a stronger electrostatic driving force to overcome the binding strength, as demonstrated by our PMF calculations (Fig. 8). Therefore, multiple Ca^2+^ ions must line up and march in a concerted manner to permeate more efficiently. Consequently, multiple Ca^2+^ binding sites lead to a concerted permeation manner, which is a characteristic that discriminates the Ca^2+^ permeation in TRPV6 from TRPV1, as also observed by Ives et al. in TRPV5 (Ives et al., 2023). Ives et al. conducted MD simulations on TRPV5 and TRPV2, with distance restraints to keep the gate open and otherwise similar simulation conditions to this study. The consistent results may indicate conserved ion permeation mechanisms in the TRPV ion channel family.

In fact, increasing the number of Ca^2+^ binding sites in channel pores has long been proposed to improve channel selectivity for Ca^2+^ (Tsien et al., 1987). Under physiological conditions, this concerted permeation method only allows Na^+^ permeation when both Ca^2+^ in the SF are away, which is much rarer than the case with only one Ca^2+^ binding site. Therefore, the multiple Ca^2+^ binding sites within SF can reject Na^+^ permeation more efficiently than in TRPV1, resulting in higher Ca^2+^ selectivity. However, in our simulations with high transmembrane potential, although we observed concerted Ca^2+^ permeation, Na^+^ permeation events still occurred in TRPV6. We attribute this to the fact that a non-physiologically high transmembrane voltage (500 mV) was applied in our MD simulations to obtain sufficient statistics, which would generate a much stronger driving force for cations to permeate than under physiological conditions. Thus, monovalent cations can probably still push Ca^2+^ through SF, resulting in a lower valence selectivity than expected. Indeed, Ives et al.’s work observed similar behavior for TRPV5, as well as that the selectivity increases in TRPV5 with decreasing the transmembrane potential (Ives et al., 2023). Hence, caution should be exercised when interpreting the non-equilibrium simulation results on valence selectivity presented in this study. To further evaluate this, we conducted PMF calculations and found that the binding of an incoming ion at the upper SF would facilitate the permeation of the Ca^2+^ at the lower SF of TRPV6, and the effect is more pronounced when the incoming ion is Ca^2+^ than Na^+^. Although the PMF calculation was conducted under non-physiological conditions as well, we think the difference in binding strength reduction of ∼25 kJ/mol is quantitatively informative. This supports the notion that successive occupation of Ca^2+^ in the SF would be more efficient for continuous ion permeation. Nonetheless, we should also point out the possibility that our non-polarizable multisite Ca^2+^ model, although optimized for calculating ion–protein interactions and working well for wide channels like RyRs (Zhang et al., 2020; Liu et al., 2021), may still lack the necessary accuracy for simulating accumulated Ca^2+^ ions confined in a narrow pore. In such cases, the polarizable effect becomes more significant and difficult to account for. It would be interesting to conduct further simulation studies with polarizable force fields in the future.

Ca^2+^ permeates through the SF of both TRPV6 and TRPV1 in a slightly dehydrated manner, and dehydration occurs in a more extended region of TRPV6, suggesting that the permeation pathway is more constricted for hydrated Ca^2+^ in the SF of TRPV6 than in TRPV1. Fortunately, the dehydration of Ca^2+^ in TRPV6 occurs around the charged residues in the SF and the oxygen atoms of the charged residues can take the role of coordination with the permeant Ca^2+^, thus lowering the dehydration-free energy barrier. In contrast, for TRPV1, the steric constriction site is below the electrostatic attraction site in SF. Although TRPV6 and TRPV1 utilize electrostatic and steric interactions to attract and bind Ca^2+^, they have different strategies to regulate ion permeation in the SF.

The distinct permeation mechanisms of TRPV6 and TRPV1 are based on their distinct SF structures. In the Ca^2+^-selective channel TRPV6, two adjacent binding sites line up in the narrow and long SF regions, consisting of negatively charged D542 and polar T539. This SF structure, which is ∼2 nm in length, enables Ca^2+^ to bind partially dehydrated and allows two Ca^2+^ ions to bind simultaneously in the SF region. In contrast, the SF of TRPV1 is much shorter and has a wide vestibule of negative electrostatic potential above the SF, with the narrowest region consisting of only one non-charged G643. This structure only allows for one Ca^2+^ binding site, and Ca^2+^ in the upper vestibule is dynamic and less efficient in blocking the permeation of Na^+^.

Although conventionally called knock-off (Hess and Tsien, 1984; Tang et al., 2014), the concerted Ca^2+^ permeation through multiple binding sites in TRPV6 is similar to the knock-on permeation behavior in the selective Na^+^ and K^+^ channels, which possess two Na^+^ and four K^+^ binding sites in the SF region, respectively (Corry and Thomas, 2012; Köpfer et al., 2014). However, there are evident differences in the selectivity mechanisms of these channels. In the selective Ca^2+^ channels, such as TRPV6, slightly dehydrated Ca^2+^ ions occupy two binding sites in the SF that are farther away from each other (with a separation of 11 Å), and the determinative factor for ion selectivity is likely the difference in electrostatic binding affinity and driving force generated by different valences. In this context, we can probably say that the Ca^2+^ knock-off was achieved by multiple Ca^2+^ ions’ remote knock-on. In the highly selective K^+^ channels, fully dehydrated K^+^ ions sit next to each other (with a separation of about 3.4 Å), and the determinant factor for ion selectivity is the dehydration energy difference of various ions when entering the SF. The highly selective Na^+^ channels also showed two loosely packed Na^+^ binding sites in the SF (with a separation of 10 Å), where the determinative factor was the partial dehydration energy when Na^+^ entered the SF. In fact, the difference in the (partial) dehydration energy of K^+^ and Na^+^ entering the SF can also be viewed as the difference in the binding affinities of these monovalent ions to the SF.

Therefore, by studying two representative TRPV channels with distinct Ca^2+^ binding and permeation patterns, as well as relating the results to the existing knowledge of highly selective Na^+^ and K^+^ channels, we conclude that ion binding competition at multiple binding sites in the SF of ion channels is required to generate high-blocking effect or ion selectivity. Ion competition at one binding site can generate a moderate blocking effect and ion selectivity. To achieve high blocking or selectivity while maintaining ion permeation, multiple ion binding sites with the same ion preference in SF are required. In such a scenario, the permeating ions would have to follow a concerted knock-on permeation behavior, which can simultaneously facilitate optimal ion permeability and selectivity. In addition, our results showed that not only the specific state of the channel structure but also the flexibility of the SF is essential for regulating ion permeability, highlighting the importance of protein dynamics.

It should be kept in mind, though, that our simulations were performed under non-physiological conditions (high [Ca^2+^] and high voltage), and therefore some of the results may not be quantitatively accurate. For instance, the selectivity may be transmembrane potential dependent, and a non-physiologically high voltage would abolish valence selectivity as observed in our simulations. Therefore, our conclusions on ion binding and permeation are probably more reliable than those on ion selectivity. To more quantitatively study ion valence selectivity under physiological conditions, complete ion channel structures in a realistic simulation setup, more accurate ion models that better consider polarization, physiological Ca^2+^ concentration and transmembrane voltage, and longer simulation time are desirable in future simulation studies.

## Supplementary Material

Table S1 shows the CHARMM-GUI equilibration protocol and parameters.

Table S2 shows restrain conditions that were tested in our MD simulations.

## Data Availability

The MD simulation input files and analysis scripts used for this study are deposited in a public GitHub repository, available at: https://github.com/songcgroup/Ca_in_TRPV. The other data are available from the corresponding author upon reasonable request.
