# Supplementary material for: Calcium binding and permeation in TRPV channels: Insights from molecular dynamics simulations
Source: J Gen Physiol. 2023 Sep 20;155(12):e202213261. doi: 10.1085/jgp.202213261 (PMC10510737; doi:10.1085/jgp.202213261)
Supplement: Table S1 — shows the CHARMM-GUI equilibration protocol and parameters. [file JGP_202213261_TableS1.docx]

**Table S1. The CHARMM-GUI equilibration protocol and parameters.**

| Steps | Time step (fs) | Simulation time (ps) | Ensemble | Restraints | | | |
| --- | --- | --- | --- | --- | --- | --- | --- |
|  |  |  |  | Protein backbone  (kJ ·mol^-1^·nm^-2^) | Protein sidechain  (kJ ·mol^-1^·nm^-2^) | P atoms in lipids  (kJ ·mol^-1^·nm^-2^) | Dihedral  (kJ ·mol^-1^) |
| 1 | 1 | 125 | NVT | 4000 | 2000 | 1000 | 1000 |
| 2 | 1 | 125 | NVT | 2000 | 1000 | 400 | 400 |
| 3 | 1 | 125 | NPT | 1000 | 500 | 400 | 200 |
| 4 | 2 | 500 | NPT | 500 | 200 | 200 | 200 |
| 5 | 2 | 500 | NPT | 200 | 50 | 40 | 100 |
| 6 | 2 | 500 | NPT | 50 | 0 | 0 | 0 |
