# Supplementary material for: Calcium binding and permeation in TRPV channels: Insights from molecular dynamics simulations
Source: J Gen Physiol. 2023 Sep 20;155(12):e202213261. doi: 10.1085/jgp.202213261 (PMC10510737; doi:10.1085/jgp.202213261)
Supplement: Table S2 — shows restrain conditions that were tested in our MD simulations. [file JGP_202213261_TableS2.docx]

**Table S2.** **Restrain conditions that were tested in our MD simulations.**

| Restrain Condition | Restrain Type | Restrain Atoms | | Force  Constant |
| --- | --- | --- | --- | --- |
|  |  | TRPV6 | TRPV1 |  |
| Restrained Protein | Position | All α-carbon | All α-carbon | 1000  kJ mol^−1^ nm^−2^ |
| Flexible SF |  | All α-carbon except SF (residue 539-542) | All α-carbon except SF (residue 643-656) |  |
| Flexible Pore Helix |  | All α-carbon except SF and pore helix  (residue 514-552) | All α-carbon except SF and pore helix (residue 600-656) |  |
| Flexible Loop and Pore Helix* |  | All α-carbon except loops between the transmembrane helices and pore helix regions (residue 350-378, 405-424, 447-449, 470-475, 514-552) | All α-carbon except loops between the transmembrane helices and pore helix regions (residue 456-468, 501-510, 533-537, 557-561, 600-656) |  |

* This is the condition for the production simulations.
